# The mitochondrial genome of the Jeju ground beetle *Carabus smaragdinus monilifer* (Coleoptera, Carabidae) 

**DOI:** 10.1080/23802359.2019.1692708

**Published:** 2019-12-09

**Authors:** Dae-Ju Oh, Kyoung-Sik Yang, Yong-Hwan Jung

**Affiliations:** Biodiversity Research Institute, Jeju Technopark, Seogwipo, Republic of Korea

**Keywords:** Carabus, mitochondrial genome, phylogeny

## Abstract

The complete mitochondrial genome of the Jeju ground beetle *Carabus smaragdinus monilifer* was analyzed to determine its structure, morphology, and other characteristics. The 16,737-bp long mitochondrial genome consisted of 37 genes, including 13 protein-coding genes, two rRNAs, and 22 tRNAs. The order, encoding direction, and the initiation and termination codons of the 37 genes of *C. smaragdinus monilifer* were identical to those of other species in the family Carabidae. Phylogenetic analysis revealed that *C. smaragdinus monilifer* is clustered with *Carabus lafossei*. Herein, we have provided the complete mitochondrial genome sequence of *C. smaragdinus monilifer* to understand the phylogeny of Carabidae.

The carabid beetles belong to the family Coleoptera, which is one of the most diverse taxa. The ground beetle *Carabus smaragdinus* is classified into 11 subspecies in Korea. *C. smaragdinus monilifer* is the first recorded species from the Korean peninsula, and was found in Jeju Island by Tatum in 1847.

Despite the vast diversity of insect species, information about their phylogeny is lacking. Several researchers have used mitochondrial (mt) genomes to study molecular phylogeny (Wan et al. 2012; Cameron 2014; Kim et al. 2017).

Of the 39,819 species of the family Carabidae that have been reported (http://carabidae.org), only 8 mt genome sequences have been deposited in the GenBank database. Thus, we sequenced the mt genome of *C. smaragdinus monilifer* (Specimen num. JBRI-INSCT-0122) of the Jeju Island (geospatial coordinates: 33°18′N 126°38′E) and analyzed its structure, morphology, and other characteristics. Total DNA was extracted and stored in deep freezer in the Jeju Biodiversity Research Institute.

The 16,737-base pairs long mt genome of *C. smaragdinus monilifer* was deposited in GenBank (accession no. MN480425), and consisted of 37 genes, including 13 protein-coding genes (PCGs), two rRNAs, and 22 tRNAs. The mt gene order of *C. smaragdinus monilifer* was identical to that of other ground beetles. Moreover, similar to other ground beetles, most protein-coding genes and tRNAs were encoded on the H-strand, and four PCGs (NADH dehydrogenase subunit 1, 4, 4 L, and 5) and eight tRNAs (G, C, Y, P, H, P, L, and V) were encoded on the L-strand. The 16S and 12S ribosomal RNA were also encoded on the L-strand.

All PCGs were initiated by ATT and ATG except NADH dehydrogenase subunit 1, which was initiated by TTG. All PCGs were terminated by TAA, TAG, and the incomplete termination codon T.

The mt genome of *C. smaragdinus monilifer* contained three tRNA clusters (IQM, WCY, and ARNSEF) that were conserved in the mt genome of other ground beetles (Song et al. 2010; Wan et al. 2012; Liu et al. 2018; Wang et al. 2018).

The phylogenetic relationship between *C. smaragdinus monilifer* and eight other Carabidae species previously reported in the GenBank database was determined using the newly determined mt genome sequences of *C. smaragdinus monilifer*. The maximum likelihood (ML) tree was constructed with 13 PCG sequences (Figure 1).

**Figure 1.**
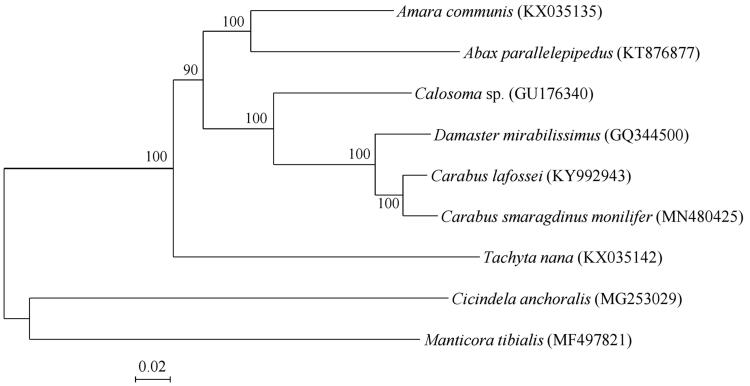
Molecular phylogenetic analysis of mitochondrial 13 protein-coding genes in 9 species of Carabidae. The phylogenetic tree was constructed using MEGA X software (Kumar et al. 2018) with maximum likelihood method using the GTR + G + I model. Percentages of trees where associated taxa were clustered together were shown next to branches.

The ML tree revealed that *C. smaragdinus monilifer* is clustered together with *Carabus lafossei*. However, phylogenetic analysis could not provide much information because only eight mt genomes in the Carabidae have been reported in the GenBank database.

Several studies have been conducted to investigate the phylogenetic status and evolutionary history of the Coleoptera mt genes (Juan et al. 1996; Clark et al. 2001; Sota and Ishikawa 2004; Ribera et al. 2004; Andújar et al. 2012; Deuve et al. 2012; Yuan et al. 2016; Linard et al. 2018). Although Carabidae is one of the largest insect families, the mt genome sequences of its species are poorly reported. Thus, in this study, we have provided the mt genome of *C. smaragdinus monilifer* to understand the molecular phylogeny of the family Carabidae.
